# Symptomatic chikungunya and chronic post-infection arthralgia in a highly endemic setting in Northeastern Brazil, 2018–2019: Clinical characteristics, prevalence and associated factors

**DOI:** 10.1371/journal.pone.0328141

**Published:** 2026-01-13

**Authors:** Carolline A. Mariz, Natália Menezes N. de Oliveira, Sílvia Carla de A. Alexandre, Isabelle Viana, Clarice N. L. de Morais, Ernesto T. A. Marques, Thomas Jaenisch, Wayner Vieira de Souza, Maria de Fátima P. Militão de Albuquerque, Carlos A. A. de Brito, Cynthia Braga

**Affiliations:** 1 Department of Parasitology, Institute Aggeu Magalhães, Oswaldo Cruz Foundation, Recife, Pernambuco, Brazil; 2 Universidade de Pernambuco, Recife, Pernambuco, Brazil; 3 Department of Virology, Institute Aggeu Magalhães, Oswaldo Cruz Foundation, Recife, Pernambuco, Brazil; 4 Department of Infectious Diseases and Microbiology, University of Pittsburgh, Pittsburgh, Pennsylvania, United States of America; 5 Section Clinical Tropical Medicine, Department of Infectious Diseases, Heidelberg University Hospital, Germany; 6 German Centre for Infection Research (DZIF), Heidelberg Site, Heidelberg, Germany; 7 Center for Global Health, Colorado School of Public Health, Aurora, Colorado, United States of America; 8 Department of Public Health, Institute Aggeu Magalhães, Oswaldo Cruz Foundation, Recife, Pernambuco, Brazil; 9 Department of Clinical Medicine, Federal University of Pernambuco, Recife, Pernambuco, Brazil; 10 Department of Immunology, Autoimune Research Institute, Recife, Pernambuco, Brazil; Instituto Nacional de Salud Publica Centro de Investigaciones sobre Enfermedades Infecciosas, MEXICO

## Abstract

Chikungunya, an *Aedes*-borne disease, poses a significant global health threat due to its substantial morbidity. The prevalence of symptomatic chikungunya virus (CHIKV) infection and chronic arthralgia, as well as their associated factors, vary geographically and across studies. We estimated the prevalence of these outcomes in a household-based survey conducted in a large northeastern Brazilian city (2018–2019) approximately three years after the city’s first CHIKV outbreak (2016). Sociodemographic and clinical data were collected through interviews, and arboviruses serostatus (IgG and/or IgM) was determined using ELISA. Arthralgia severity was assessed via Visual Analog Scale (VAS). Prevalence estimates (95% CI) and adjusted prevalence ratios (aPRs) were estimated using Poisson regression with robust variance. Principal Component Analysis (PCA) was used to address multicollinearity and identify latent risk profiles . Of the 760 CHIKV-exposed participants, 70% (95% CI: 66.7–73.2; n = 532) reported symptomatic infections. Among those reporting arthralgia (n = 499), 36.5% (95% CI: 32.4–40.8) experienced symptoms lasting >90 days, and of these, over 70% reported severe pain (VAS ≥ 8). In multivariable analyses, older age (aPR = 1.21 [36–50 years], aPR = 1.19 [51–65 years]), female sex (aPR = 1.19; 95% CI: 1.08–1.32), and prior DENV exposure (aPR = 1.45; 95% CI: 1.03–2.04) were associated with – symptomatic infection, whereas higher income showed a protective association. PCA confirmed these formed distinct risk profiles; a sociodemographic component (older age, prior DENV and single marital status) and a biological sex component each independently increased the odds of symptomatic disease by 31% (aOR=1.31). For chronic arthralgia, risk increased with older age (aPR = 4.60 [51–65 years]), female sex (aPR = 1.70; 95% CI: 1.29–2.25), and severe acute pain (aPR = 2.91; 95% CI: 1.86–4.55), but inversely associated with low income (aPR = 0.67). These findings underscore the need for targeted interventions, particularly for older adults, women, and low-income groups. Further studies are needed to elucidate the immunological mechanisms underlying these associations.

## Introduction

Chikungunya, a mosquito-borne disease primarily transmitted by Aedes aegypti and Aedes albopictus, has emerged as a significant global health problem, particularly in Asian, African and the Americas [1,2]. Between 2011 and 2020, an estimated 19 million cases were reported worldwide, with approximately 80% occurring in Latin America and the Caribbean region [2].

Infection by the chikungunya virus (CHIKV, family *Togaviridae*, genus *Alphavirus*) has a wide spectrum of manifestations, ranging from asymptomatic or mild cases to severe and debilitating conditions, including neurological disorders and chronic arthralgia [3]. The prevalence of symptomatic chikungunya varies significantly across regions and populations, with an estimated rate of 75% (range: 63%−84%) [4]. The acute phase of chikungunya is primarily characterized by high fever (>39ºC), rash, headache, arthralgia and myalgia, symptoms that overlap with other arboviral infections, such as dengue [4].

Arthralgia is one of the most prominent symptoms of chikungunya, occurring in approximately 90% of cases. It is typically bilateral and symmetrical, predominantly affecting the joints of the hands, feet, knees and wrists [4,5]. Persistent arthralgia, defined as joint pain lasting beyond three months after the acute phase, is a major complication, affecting 30–60% of infected individuals [6–8]. A systematic review of 67 studies estimated the prevalence of chronic arthralgia at 44% after three months, declining to 34% after six months and 32% at twelve months [4].

The frequency of symptomatic chikungunya and persistent arthralgia post-infection, as well as the factors associated with these events, has shown high geographic heterogeneity and variation between studies [4,7]. Both events have been linked to a number of individual factors, including sociodemographic characteristics (e.g., advanced age and female sex) [9,10], comorbidities (diabetes, hypertension, ischemic heart disease, pre-existing joint disease), genetic polymorphisms and immunological factors [7,11–13]. Viral strain-related factors, such as the CHIKV lineage, have also been associated with chronic arthralgia. Specifically, the Asian lineage has been associated with a higher incidence of inapparent chikungunya infections [14], whereas the Indian Ocean lineage (a sublineage of the East/Central/South African [ECSA] strain) has been linked to chronic arthralgia [15].

Since its first detection in Brazil in 2014, CHIKV has caused severe recurrent epidemics nationwide. From 2013 to 2022, seven epidemic waves were recorded, with the Northeast region accounting for ~65% of confirmed cases being among the most affected [16]. To evaluate the long-term transmission patterns of CHIKV and other arboviruses, we conducted a population-based seroprevalence survey in Recife (2018–2019), a major urban center in Northeastern Brazil, three years after the city’s first major CHIKV epidemic (2016) [17]. The results showed an overall CHIKV, DENV and Zika (ZIKV) seroprevalence of 35.7%, 88.7% and 37.2%, respectively. Based on the estimated CHIKV seroprevalence estimate (35.7%, n = 2.070), we extrapolated that approximately 600.000 residents were infected between the virus’s emergence in 2015 and 2018/2019 survey period [17]. In this study, we estimated the prevalence of symptomatic CHIKV infection and persistent post-infection arthralgia, as well as investigated associated risk factors among the CHIKV-exposed participants in this survey.

## Materials and methods

### Design, setting and study population

The multistage stratified cluster sampling population survey was conducted among residents aged 5–65 years between August 2018 and February 2019. Details on the sampling methods and selection of study participants have been described elsewhere [17]. All survey participants with positive serology for CHIKV were included in this study (IgM/IgG; n = 760).

Recife, the capital of Pernambuco state, has a population of approximately 1.5 million inhabitants and one of Brazil’s highest population densities (7,000 inhabitants/km²) [18].

Since the first dengue epidemic in the 1980s, the city has experienced recurrent arbovirus outbreaks. Population-based studies conducted in the city prior to the introduction of ZIKV and CHIKV demonstrated exceptionally high dengue seroprevalence. A 2005–2006 survey of 2.833 residents from distinct socioeconomic areas found a dengue seroprevalence of 74–91% [19]. Subsequent analysis of a subset (n = 323) of DENV-positive individuals revealed antibodies to DENV-3 (93.2%), DENV-1 (53.8%), and DENV-2 (32.2%), indicating a historical pattern of exposure dominated by DENV-3 [20]. These findings were confirmed by another ZIKV and CHIKV pre-epidemic study (2011–2012) of healthy pregnant women in the same region, which showed a dengue seroprevalence of 95% [21]. During the subsequent ZIKV/CHIKV epidemic periods, hospital-based studies confirmed high DENV population-level immunity, alongside low rates of active DENV infection confirmed by RT-PCR or seroconversion [22,23].

### Data collection

In the serosurvey, we invited all eligible residents of selected households within the study’s age range to participate. Participants read and signed the Informed Consent Form before study staff collected sociodemographic and clinical data through interviews using a structured questionnaire. For participants with arthralgia, pain intensity was assessed using a visual analog scale (VAS; 0 = no pain, 10 = very severe pain) [18]. Subsequently, a venous blood sample was collected for serological testing. Further detail on data collection was previously described [17].

### Laboratory procedures

Detection of anti-CHIKV IgG and IgM antibodies was performed using commercial ELISA kits (Euroimmun, Lubeck, Germany) and the results were interpreted according to the manufacturer’s instructions (CHIKV IgG or IgM absorbance at 450 nm/calibrator ratios were considered negative at <0.8, indeterminate at ≥0.8 to <1.1, and positive at ≥1.1). Previous or recent exposure to ZIKV was assessed by detecting IgG ELISA (Euroimmun, Lubeck, Germany) and by detecting IgG3 antibodies against the ZIKV NS1 protein through an in-house ELISA [17]. Anti-DENV IgG was determined by an in-house indirect ELISA method, as described elsewhere [24]. Results were interpreted as positive when the sample absorbance at 450 nm/calibrator ratio ≥3.62.

### Case definitions

Symptomatic chikungunya: defined as laboratory detection of anti-CHIKV antibodies (IgG and/or IgM) accompanied by one of the following conditions:

(1) A self-reported history of Chikungunya within the preceding three years; or
(2) No self-reported history of Chikungunya but self-reported dengue or Zika in the previous three years, with negative serology for ZIKV (anti-ZIKV IgG, IgM, and/or IgG3).

This second case definition criterium was established due the significant clinical overlap between arboviral infections (dengue, Zika, and chikungunya); the recent and near-simultaneous introduction of ZIKV and CHIKV in the study region [20]; and evidence of low DENV circulation during survey data collection [22,23].

Chronic arthralgia: defined as the report of joint pain persisting for >90 days after the acute phase of infection.

### Exposed variables

Demographic: Age group (5–19, 20–35, 36–50, and 51–65 years), sex, self-reported race/skin color (brown, black, white, and asian), educational level (elementary, high school, university/postgraduate), marital status (with or without partner), head of family income in minimum wages (≤2, > 2 to ≤4, and >4).

Clinical: Comorbidities (hypertension, diabetes, chronic heart disease, nephropathy, or others), DENV immune status (anti-DENV IgG), joint pain intensity (assessed by visual analogue scale, VAS [1–10 score]), severity of joint pain during acute phase of the disease (very severe = VAS > 8), self-reported chikungunya symptoms (fever [and duration], headache, rash, conjunctivitis, photophobia, myalgia, eye pain, arthralgia, edema, anorexia, prostration, bleeding, limb paralysis, meningitis, pruritus, diarrhea, and nausea/vomiting), as well as joints affected by pain.

### Data analysis

Data management was performed using the REDCap electronic platform (hosted at the University of Heidelberg, Germany), and statistical analyses were conducted using Stata software (version 15; StataCorp, College Station, TX, USA).

We first characterized the study population by describing the frequency distributions of key variables, presenting means ± standard deviations for continuous variables and proportions for categorical variables. Difference between means were assessed by Kruskal Wallis (KW) test (p-value<0.05). Prevalence estimates with 95% confidence intervals (95% CI) were calculated for symptomatic chikungunya and persistent arthralgia. The data analysis of chronic arthralgia was restricted to symptomatic cases (n = 532).

To assess associations between exposure variables and the clinical outcomes (symptomatic chikungunya or chronic arthralgia), we estimated both crude (PR) and adjusted prevalence ratios (aPRs) using Poisson regression with robust variance. This method is preferable for estimating association measures for high-prevalence outcomes [25]. To explore potential underlying latent structures and reduce dimensionality among the set of variables associated with the outcomes (p-value<0.20), we also conducted Principal Component Analysis (PCA).

For the symptomatic chikungunya model, the PCA-derived components were analyzed using logistic regression to estimate adjusted Odds Ratios (aORs). This approach was chosen to enable robust goodness-of-fit validation (e.g., Hosmer-Lemeshow test). A sensitivity analysis using Poisson regression with robust variance yielded qualitatively similar results, confirming the consistency of the identified associations.

Initially, we examined the unadjusted associations between a range of sociodemographic (age group, sex, self-reported race/skin color, educational level, marital status, head of family income) and clinical factors (comorbidities, DENV-immune status, severity of joint pain) and each outcome of interest (symptomatic chikungunya or chronic arthralgia). Separate analyses were conducted for symptomatic infection and for chronic arthralgia. The severity of joint pain was analyzed exclusively in the model for chronic arthralgia.

Variables associated with the outcomes at p < 0.20 in univariable analyses were selected for inclusion in the multivariable regression model. Variables significantly associated with the outcome (p < 0.05) were retained in the final multivariable model.

For the chronic arthralgia outcome, the PCA results indicated that this technique was not suitable. This was evidenced by a low Kaiser-Meyer-Olkin measure of sampling adequacy (KMO = 0.5068) and insufficient variable communalities (range: 0.010–0.307), suggesting that the set of variables did not share enough common variance to form reliable components (S1 File). Therefore, we maintained multiple Poisson regression as our primary analytical approach. To ensure the robustness of our regression estimates, we performed comprehensive sensitivity analyses including multiple imputations for missing data.

### Ethical issues

This study was approved by the Research Ethics Committee of the Aggeu Magalhães Institute (IAM/Fiocruz, Pernambuco) (CAEE: 79605717.9.0000.5190; approval number: 2.734.481). Research data were collected after participants (or their legal guardians if under 18) were informed about the study’s objectives and provided written informed consent, in accordance with Brazilian National Health Council Resolution No. 466/12. Participants aged 5–18 also provided oral and/or written assent.

## Results

A total of 760 survey participants were exposed to CHIKV. The median age was 38 years (range: 5–65), with a predominance of females (60.3%, 458/760), 54% (411/760) self-identifying as mixed-race (brown skin color) and 30.7% (175/760) reporting comorbidities. The seroprevalence of infection was 93.8% for DENV (n = 713; 95% CI: 91.9–95.3%) and 50.2% for Zika virus (n = 384; 95% CI: 46.9–54.1%) in the study population.

Of these, 532 met the symptomatic case definition criteria, resulting in a symptomatic chikungunya prevalence of 70% (95% CI: 66.7–73.2) and a symptomatic-to-asymptomatic ratio (SAR) of 2.16:1 (95% CI: 1.89:1–2.37:1).

The most frequently reported symptoms among symptomatic cases were arthralgia (93.8%), myalgia (91.3%), fever (89.8%), prostration (89.8%), and headache (81.4%). Rash, photophobia, eye pain, and edema were reported in approximately half of cases (Table 1).

**Table 1:** Frequency distribution of the reported symptoms among cases of symptomatic chikungunya. Recife, Northeast Brazil, 2018-2019.

Characteristics/symptoms	n = 532
Arthralgia	499 (93.8)
Myalgia	486 (91.3)
Severe/very severe myalgia, n (%)	341 (70.6)
Fever, n (%)	478 (89.8)
Duration of fever (in days), mean (Standard deviation, SD)	4.7 (4)
Prostation, n (%)	478 (89.8)
Headache, n (%)	433 (81.4)
Anorexia, n (%)	421 (79.1)
Severe/very severe headache (VAS* > 8)	221 (51.2)
Rash, n (%)	293 (55.1)
Photophobia, n (%)	282 (53.0)
Eye pain, n (%)	298 (56.0)
Conjunctivitis, n (%)	200 (37.6)
Edema, n (%)	246 (46.3)
Bleeding, n (%)	4 (0.7)
Paralysis of limbs, n (%)	16 (3.0)
Meningitis, n (%)	2 (0.4)
Itching, n (%)	97 (18.2)
Diarrehea, n (%)	35 (4.6)
Nausea/vomit, n (%)	99 (13.0)
**Characteristics of arthralgia**	n = 499
Intensity of pain, mean (SD) (VAS*)	8.3 (1.9)
Severe/very severe arthralgia, n (%) (VAS ≥ 8)	361 (72.3)
**Joints affected, n (%)**	
Hands	429 (89.9)
Wrist	431 (86.4)
Foot	424 (84.9)
Ankles	419 (83.9)
Knees	406 (81.4)
Elbows	312 (62.5)
Shoulders	323 (64.7)

* Visual analog scale.

Among participants reporting arthralgia (n = 499), over 70% experienced severe pain (VAS score ≥ 8), with a mean pain intensity of 8.3 ± 1.9 (median 9: range 1–10). The most frequently affected joints were those of the hands (89.9%), wrists (86.4%), feet (84.9%), ankles (83.9%), and knees (81.4%). Elbows and shoulders were involved in over half of cases (Table 1).

Pain intensity was significantly higher in those with arthralgia persisting >90 days (mean ± SD: 9.1 ± 1.5) compared to those with shorter symptom duration (7.9 ± 2.0; KW χ² = 54.61, p < 0.001). Similarly, females reported higher pain intensity than males (8.7 ± 0.1 vs. 7.6 ± 0.2; KW χ² = 30.37, p < 0.001), as did participants with comorbidities compared to those without (8.6 ± 0.15 vs. 8.2 ± 0.11; KW χ² = 6.71, p = 0.009).

In the univariable regression analysis, age group, sex, marital status, head of household income, education level, comorbidities, and prior DENV exposure (IgG anti-DENV) were associated with symptomatic chikungunya (p < 0.20) and were retained for multivariable analysis (Table 2). To address potential multicollinearity and explore latent structures among these variables, we performed a PCA. The data were suitable for PCA (Bartlett’s test: χ²(15) = 441.79, p < 0.001; KMO = 0.65).

**Table 2:** Crude and adjusted prevalence rates (PR) of the association of sociodemographic and clinical characteristics with symptomatic chikungunya. Recife, Northeast Brazil, 2018-2019.

	Positives (n = 760)	Symptomatic (%)	Crude PR (95 CI%)	p-valor	Adjusted PR (IC95%)	p-valor
**Age (years)**						
5–19	147	85 (57.8)	1.00		1.00	–
20–35	194	128 (66.0)	1.14 (0.96 - 1.35)	0.131	1.05 (0.88–1.24)	0.582
36–50	204	158 (77.5)	1.34 (1.14–1.57)	<0.001	1.21 (1.03–1.41)	0.017
51–65	215	161 (74.9)	1.29 (1.11–1.52)	0.001	1.19 (1.01–1.40)	0.035
**Sex**						
Male	302	187 (61.9)	1.00	–	1.00	–
Female	458	345 (75.3)	1.22 (1.10–1.35)	<0.001	1.19 (1.08–1.32)	0.001
**Self-reported race/skin color**						
Mixed	411	284 (69.1)	1.00			
Black	133	98 (73.7)	1.07 (0.95–1.20)	0.296	–	
White	209	144 (69.0)	1.00 (0.90–1.12)	0.959		
Asiatic	7	4 (85.7)	1.24 (0.91–1.70)	0.172		
**Comorbidity**						
No	527	357 (67.7)	1.00		**–**	
Yes	233	175 (75.1)	1.11 (1.01–1.22)	0.032		
**Prior DENV exposure (DENV IgG ELISA)**						
Negative	47	20 (42.6)	1.00		1.00	
Positive	713	512 (71.8)	1.69 (1.21–2.36)	0.002	1.45 (1.03–2.04)	0.032
**Marital status (with partner)** ^a^						
No	368	276 (75.0)	1.00			
Yes	329	224 (68.1)	0.95 (0.92–0.98)	0.002		–
**Schooling** ^**c**^						
University/Posgraduated	125	84 (67.2)	1.00		–	
Fundamental	288	191 (66.3)	0.98 (0.85–1.14)	0.861		
High school	323	245 (75.9)	1.13 (0.98–1.30)	0.084		
**Head of household income (minimum wage)** ^**b**^						
> 4	74	39 (52.7)	1.00		1.00	
> 2 e ≤ 4	149	109 (73.2)	1.39 (1.10–1.76)	0.007	1.37 (1.08 −1.73)	0.008
≤ 2	515	370 (71.8)	1.36 (1.10–1.70)	0.006	1.34 (1.08–1.67)	0.009

^a^ 63 not applicable (age < 13 yrs); ^b^ 22 missing records; ^c^ 24 missing records

A three-component structure, explaining 66.6% of the total variance, was identified: Component 1 (Sociodemographic Factor), that explained 31.4% of the variance and characterized by positive loadings for older age (0.55), prior DENV exposure (0.56), and a negative loading for being married/in a union (−0.62); Component 2 (Socioeconomic Status Factor), that explained 18.6% of the variance, was defined by a positive loading for higher income (0.73) and a negative loading for higher education level (−0.67) and; Component 3 (Biological Sex Factor), explaining 16.7% of the variance, that loaded almost exclusively on female sex (0.998).

In a multivariable logistic regression analysis model, both Components 1 and 3 were significantly independent predictors of symptomatic chikungunya. A one-standard-deviation increase in Component 1 was associated with 31% higher adjusted odds of symptomatic infection (aOR = 1.31, 95% CI: 1.17–1.46, p < 0.001). Similarly, Component 3 was associated with 34% higher odds per one-standard-deviation increase (aOR = 1.34, 95% CI: 1.15–1.57, p < 0.001). Component 2 was not significantly associated with the outcome (aOR = 1.16, 95% CI: 0.99–1.36, p = 0.062). This model showed acceptable goodness-of-fit (Hosmer-Lemeshow test: χ²(8) = 8.11, p = 0.423) and correctly classified 71.5% of cases (S1 File).

Following, a multivariable Poisson regression was performed to estimate the independent effect of each variable. The analysis confirmed that older age (≥36 years; aPR = 1.21 for 36–50 years and 1.19 for 51–65 years), female sex (aPR = 1.19; 95% CI: 1.08–1.32) and prior DENV exposure (aPR = 1.45; 95% CI: 1.03–2.04) were independently associated with an increased risk for symptomatic chikungunya. Conversely, higher household income was protective, with lower income categories showing significantly higher risk (≤2 wages: aPR = 1.34; > 2–4 wages: aPR = 1.37) (Table 2).

We next examined factors associated with chronic arthralgia (>90 days) among those who were symptomatic. From 499 symptomatic cases with arthralgia, 182 (36.5%; 95% CI: 32.4–40.8) reported chronic symptoms (>90 days). Variables associated with the outcome in univariable analysis (p-value <0.20) were age group, sex, head of the household income, comorbidities and severe acute joint pain (VAS ≥ 8) and were included in the multivariable model.

To address potential bias from missing data in head of the household income variable (n = 14, 2.8%), we performed multiple imputations using chained equation (MICE), generating five imputed datasets. The high quality of the imputation was supported by a low Fraction of Missing Information (FMI = 0.062) and a low Relative Variance Increase (RVI = 0.014). Poisson regression models were run on each imputed dataset and results were pooled for the final analysis.

In the adjusted model, older age, female sex, and severe joint pain during the acute phase of disease were independently associated with an increased risk of chronic arthralgia (Table 3). A dose-response association was observed for age compared to the reference group (5–19 years); the aPR was 2.39 (20–35 years), aPR = 4.10 (36–50 years), and aPR = 4.60 (51–65 years). Female sex was associated with 70% higher prevalence (aPR = 1.70; 95% CI: 1.29–2.25), and severe acute joint pain was associated with nearly three-fold higher risk (aPR = 2.91; 95% CI: 1.86–4.55). Conversely, a lower household income (≤2 minimum wages) was associated with a 33% lower prevalence of chronic arthralgia compared to the highest income group (aPR = 0.67; 95% CI: 0.50–0.90). Comorbidities (e.g., hypertension/diabetes) were associated with chronic arthralgia in unadjusted analysis (PR = 1.64, p < 0.01) but not in the adjusted model (Table 3).

**Table 3:** Crude and adjusted prevalence rates (PR) of the association of sociodemographic and clinical characteristics with chronic arthralgia among symptomatic cases of chikungunya (n = 499). Recife, Northeast Brazil, 2018-2019.

	Symptomatic cases reporting arthralgia	Chronic arthralgia (>3 months)	Crude RP (95% CI)	p-valor	Adjusted PR (95% CI)	p-valor
**Age (years)**						
5–19	68	6 (8.8)	1.00		1.00	
20–35	123	31 (25.2)	2.86 (1.25–6.51)	0.012	2.39 (1.06–13.40)	0.037
36–50	152	67 (44.1)	4.99 (2.28–10.96)	<0.001	4.10 (1.87–8.97)	<0.001
51–65	156	78 (50.0)	5.67 (2.60–12.40)	<0.001	4.60 (2.10–10.03)	<0.001
**Sex**						
Male	169	42 (24.9)	1.00	–		
Female	330	140 (42.4)	1.71 (1.28–2.28)	<0.001	1.40 (1.07–1.83)	0.015
**Self-reported race/skin color**						
Mixed	271 (54.3)	101 (37.3)	1.00		–	
Black	90 (18.0)	30 (33.3)	0.89 (0.64–1.25)	0.508		
White	132 (26.5)	50 (37.9)	1.02 (0.78 - 1.32)	0.906		
Asiatic	6 (1.2)	1 (16.7)	0.45 (0.74–2.70)	0.380		
**Marital status (with partner)** ^a^						
No	265	110 (41.5)	1.00		–	
Yes	210	68 (32.4)	0.88 (0.77–0.99)	0.038		
**Schooling** ^**c**^						
University/Posgraduated	80	34 (42.5)	1.00		**–**	
High school	236	83 (35.2)	0.86 (0.62–1.19)	0.366		
Fundamental	172	63 (36.6)	0.82 (0.61–1.13)	0.299		
**Comorbidity** ^**d**^						
No	330	99 (30.0)	1.00		–	
Yes	169	83 (49.1)	1.64 (1.30–2.05)	<0.001		
**Severe joint pain (VAS ≥ 8)**						
No	138	18 (13.0)	1.00		1.00	
Yes	361	164 (45.4)	3.48 (2.23–5.44)	<0.001	2.91 (1.86–4.55)	<0.001
**IgG anti-DENV (ELISA)**						
Negative	17	4 (23.5)	1.00			
Positive	482	178 (36.9)	1.57 (0.66–3.73)	0.308	–	
**Head of household income (minimum wage)** ^b^						
> 4	36	20 (55.6)	1.00		1.00	
> 2 e ≤ 4	102	42 (41.2)	0.74 (0.51–1.08)	0.116	0.82 (0.59–1.15)	0.244
≤ 2	347	116 (33.4)	0.60 (0.43–0.84)	0.002	0.67 (0.50–0.90)	0.008

^a^ 24 not applicable (age < 13 yrs); ^b^ 14 missing records; ^c^11 missing records; ^d^ hypertension and/or diabetes and/or heart disease and/or kidney disease.

## Discussion

In this study, we estimated the prevalence and analyzed factors associated with symptomatic chikungunya and chronic arthralgia in a population living in a hyperendemic arbovirus region, as evidenced by the high seroprevalence of DENV (93.8%) and ZIKV (50.2%).

Consistent with previous studies [4,26], the study showed a high burden of chikungunya morbidity, with 70% of study participants meeting criteria for symptomatic infection (symptomatic-to-asymptomatic ratio = 2.16:1) and a concerningly high prevalence (around 40%) of chronic arthralgia (>90 days), underscoring the significant long-term impact of CHIKV. In line with well-established clinical patterns [4,13,27], the most frequently reported symptoms were arthralgia (93.8%), myalgia (91.3%), fever (89.8%), and prostration (89.8%), with small joint, particularly the hands, wrists, and feet, most frequently affected.

Notably, over 70% of individuals who reported arthralgia in the acute phase informed severe pain, as indicated by a mean VAS score of 8.3 ± 1.9. Pain intensity was significantly higher in females, those who developed chronic arthralgia, and in individuals with comorbidities, which are groups previously established as being at risk for prolonged musculoskeletal symptoms [7,28]. Few studies have assessed joint pain intensity following CHIKV infection using the VAS [28–30]. While these studies reported moderate to severe, highlighting the clinical significance of the disease, the mean pain scores in our study population were higher than those previously documented. For instance, two cross-sectional studies, one conducted on Réunion Island (mean VAS = 5.8 ± 2.1) [29] and another in northeastern Brazil (mean VAS = 6.5 ± 2.0) [30], both reported lower pain intensity. In contrast, a prospective study of acutely ill patients admitted to hospitals in Mexico, a population likely representing more severe disease, reported higher pain scores than those in our study [28], supporting the evidence that pain intensity may reflect disease severity.

The prevalence of symptomatic chikungunya (70%) in our study population was high, aligning with rates reported in other regions [4,26]. Globally, studies have documented substantial variability in symptomatic chikungunya prevalence [4,9,14,26,31,32], suggesting that host- and virus-specific factors may shape local epidemiological patterns. Despite this variability, only three studies have analyzed risk factors, and their findings remain inconsistent [9,14,26], highlighting a critical evidence gap.

The multivariable regression analysis identified older age (>36 years), female sex, head of household income, and DENV exposure as independent factors for symptomatic chikungunya. PCA enriched this interpretation, showing the factors clustered into three latent profiles: a Sociodemographic Factor (combined older age, DENV exposure, and single marital status; aOR=1.31 per standard deviation (SD), a Socioeconomic Factor, and a distinct Biological Sex Factor (female sex; aOR=1.34 per SD. The converged findings from Poisson regression and PCA highlighted the multifactorial nature of symptomatic CHIKV infection, mainly driven by both demographic and biological sex.

The increased risk of symptomatic chikungunya with age aligns with studies from Nicaragua [14], and Brazil [31]. This trend may be explained by immunosenescence, a higher comorbidity burden, and cumulative exposure to the virus and mosquitos’ bites [33–35]. However, our findings contrast with reports from Malaysia [9] and the U.S. Virgin Islands [26]. These discrepancies may arise from differences in host genetics [36], circulating viral lineages [14], or pain perception [37].

Similarly, 20% higher in females than males (aPR = 1.19) align with studies reporting a higher frequency and severity of acute symptoms among females during CHIKV epidemics [7,28,38]. Underlying mechanisms may involve hormone-modulate immune responses [39], and established sex differences in pain perception [40]. However, it is important to note that some studies have found no significant sex-based differences [14,26], highlighting the role of other population-specific factors.

A key and novel finding was the independent association between DENV immunity and symptomatic infection (aPR = 1.45). The PCA further integrated this effect within a broader sociodemographic context of cumulative exposure (aOR=1.31) While antibody-dependent enhancement (ADE) is a postulated mechanism [41,42], *in vitro* evidence remains inconclusive for human disease and direct epidemiological evidence is still lacking. An alternative, non-exclusive explanation is that repeated mosquito bites in high-transmission areas modulate innate immunity, altering arboviral infections outcomes [43,44]. Based on these assumptions, we propose that this association may reflect broader effects of arthropod exposure, consistent with sociodemographic profile identified, rather than solely a virus-specific interaction. However, these finding merits investigation as it has profound implications for public health in co-endemic regions.

The multivariable analysis further identified lower income (≤4 minimum wages) as an independent risk factor for symptomatic CHIKV infection. Notably, households earning less than two minimum wages showed significantly higher prevalence of symptomatic cases. This association may reflect reduced healthcare-seeking behavior due to systemic barriers in healthcare access. While several studies have established links between socioeconomic status and Aedes-borne disease incidence [45,46], research specifically examining socioeconomic factors in relation to symptomatic chikungunya remains limited. Our findings are partially consistent with a Malaysian serological survey that identified higher education levels as a protective factor for symptomatic infection [9].

For the outcome chronic arthralgia, the final multivariable model confirmed established risk factors: older age (showing a clear dose-dependent effect), female sex (aPR = 1.70) and severe acute joint pain (aPR = 2.91). The strong association between older age and chronic arthralgia aligned with the literature [6,7,37] and may be explained by age-related immune dysregulation, leading to impaired viral clearance and prolonged inflammation [34], or cumulative joint vulnerability [47]. The association with female sex (aPR = 1.40) is well-documented [28,48] and may involve hormonal modulation of inflammation [39,49], leading to stronger inflammatory reactions [49], which may worsen joint pain and swelling. Psychosocial factors could also play a role in this sex difference. Women would report symptoms more frequently and exhibit greater healthcare-seeking behavior, leading to apparently higher disease severity in studies [40,50].

The finding that severe joint pain predicted chronic arthralgia is supported by previous studies [51] and may be related to a more robust inflammatory response or viral persistence [12]. However, the cross-sectional assessment of pain in our study requires caution; recall bias may influence this association as those with persistent pain may retrospectively report their acute-phase symptoms as more severe.

Interestingly, lower household income was associated with a reduced risk of chronic arthralgia (aPR = 0.67). This counterintuitive finding, which has been reported elsewhere [7,52], may reflect sociocultural normalization of pain and under-reporting in lower-income populations. Such under-reporting could mask a greater true burden of chronic morbidity within these groups.

Although studies generally indicate an inverse relationship between socioeconomic status and chronic pain, where those of lower status experience greater pain chronicity [53], emerging evidence suggests that social exclusion may correlate with reduced pain sensitivity, often described as ‘numbing’ [54].

The cross-sectional design and reliance on self-reported symptoms and retrospectively pain scoring, that potentially introduces recall and information bias, are key limitations of this study. Lack of biomarker measurements or other clinical exams, and potential unmeasured confounding factors limit causal inference. However, major strengths included the large, population-based population sample with laboratory-confirmed serostatus for CHIKV, ZIKV, and DENV, detailed clinical characterization (e.g., VAS pain scores, joint-specific data), the use of appropriate statistical models (Poisson regression) for common outcomes [25], and the application of PCA to identify latent risk profile and mitigate multicollinearity among explanatory variables. Furthermore, the study was conducted shortly after the first CHIKV epidemic amid low active DENV transmission [22,23], reducing the risk of misclassification bias between dengue and chikungunya cases.

In conclusion, this study highlights the high morbidity burden of chikungunya in a hyperendemic urban setting and identifies key demographic, clinical, and immunological risk factors. These findings underscore the need to prioritize public health interventions for higher-risk groups, such as older adults and women, while ensuring equitable access to care for chronic disability across all socioeconomic strata. Finally, the association between DENV immunity and increased risk of symptomatic CHIKV infection warrants further longitudinal studies to monitor specific immunological and genetic markers, which are needed to elucidate the possible underlying biological mechanisms.

## Supporting information

S1 File Principal component analysis and multivariable regression for chikungunya outcomes. (PDF)
