# Supplementary material for: Symptomatic chikungunya and chronic post-infection arthralgia in a highly endemic setting in Northeastern Brazil, 2018–2019: Clinical characteristics, prevalence and associated factors
Source: PLoS One. 2026 Jan 13;21(1):e0328141. doi: 10.1371/journal.pone.0328141 (PMC12798991; doi:10.1371/journal.pone.0328141)
Supplement: S1 File — (PDF) [file pone.0328141.s001.pdf]

Principal Component Analysis Principal and Multivariable Regression for Chikungunya Outcomes

Symptomatic chikungunya

<u>Check variables and descriptive analysis</u>

describe idade4 sexo igg_denv marital_2 educacao income_chef_2

```
              storage   display    value
variable name   type    format     label      variable label
-----------------------------------------------------------------------
idade4          byte    %10.0g                idade4
sexo            byte    %10.0g                sexo
igg_denv        byte    %10.0g                igg_denv
marital_2       byte    %10.0g                marital_2
educacao        byte    %10.0g                educacao
income_chef_2   byte    %10.0g                income_chef_2
```

```
summarize idade4 sexo igg_denv marital_2 educacao income_chef_2

    Variable |        Obs        Mean    Std. Dev.       Min        Max
-------------+---------------------------------------------------------
      idade4 |        760    2.640789    1.088287          1          4
        sexo |        760    .6026316    .4896757          0          1
    igg_denv |        760    .9381579    .2410272          0          1
   marital_2 |        760    1.096053    2.131385          0          8
    educacao |        760    1.006579    1.446893          0          8
-------------+---------------------------------------------------------
income_che~2 |        760    1.782895    1.258006          0          8
```

Sampling Adequacy Test
KMO and Bartlett tests

```
factor idade4 sexo igg_denv marital_2 educacao income_chef_2 (obs=760)

Factor analysis/correlation                     Number of obs    =        760
    Method: principal factors                   Retained factors =          3
    Rotation: (unrotated)                       Number of params =         15

    --------------------------------------------------------------------------
         Factor  |   Eigenvalue   Difference        Proportion   Cumulative
    -------------+------------------------------------------------------------
        Factor1  |      1.18861      1.05691            1.3231       1.3231
        Factor2  |      0.13170      0.13054            0.1466       1.4697
        Factor3  |      0.00116      0.08593            0.0013       1.4710
        Factor4  |     -0.08477      0.02579           -0.0944       1.3766
        Factor5  |     -0.11056      0.11721           -0.1231       1.2535
        Factor6  |     -0.22777            .           -0.2535       1.0000
    --------------------------------------------------------------------------
    LR test: independent vs. saturated:  chi2(15) =  441.79 Prob>chi2 = 0.0000
```

Factor loadings (pattern matrix) and unique variances

Variable	Factor1	Factor2	Factor3	Uniqueness
idade4	0.5956	-0.0346	0.0046	0.6441
sexo	0.0766	0.0600	0.0329	0.9895
igg_denv	0.5706	0.0788	-0.0046	0.6682
marital_2	-0.6934	-0.0204	0.0045	0.5187
educacao	0.1435	-0.2442	0.0030	0.9198
income_che~2	-0.0326	0.2463	-0.0026	0.9383

estat kmo

Kaiser-Meyer-Olkin measure of sampling adequacy

Variable	kmo
idade4	0.6740
sexo	0.6814
igg_denv	0.6787
marital_2	0.6140
educacao	0.6486
income_che~2	0.5068
Overall	0.6480

pca idade4 sexo igg_denv marital_2 educacao income_chef_2, comp (3)

```
Principal components/correlation                 Number of obs    =       760
                                                 Number of comp.  =         3
                                                 Trace            =         6
    Rotation: (unrotated = principal)            Rho              =    0.6662
```

Component	Eigenvalue	Difference	Proportion	Cumulative
Comp1	1.90602	.80229	0.3177	0.3177
Comp2	1.10373	.116347	0.1840	0.5016
Comp3	.98738	.103567	0.1646	0.6662
Comp4	.883813	.228618	0.1473	0.8135
Comp5	.655195	.191327	0.1092	0.9227
Comp6	.463868	.	0.0773	1.0000

Principal components (eigenvectors)

Variable	Comp1	Comp2	Comp3	Unexplained
idade4	0.5548	-0.0030	-0.0029	.4134
sexo	0.0914	0.2340	0.9661	.002084
igg_denv	0.5366	0.1496	-0.0979	.417
marital_2	-0.6048	-0.0633	0.1031	.2878
educacao	0.1681	-0.6403	0.1226	.4788
income_che~2	-0.0430	0.7133	-0.1772	.4039

```
rotate, varimax

Principal components/correlation                 Number of obs    =        760
                                                 Number of comp.  =          3
                                                 Trace            =          6
    Rotation: orthogonal varimax (Kaiser off)    Rho              =     0.6662

    --------------------------------------------------------------------------
       Component |     Variance   Difference         Proportion   Cumulative
    -------------+------------------------------------------------------------
           Comp1 |      1.88139      .767244             0.3136       0.3136
           Comp2 |      1.11415      .112568             0.1857       0.4993
           Comp3 |      1.00158            .             0.1669       0.6662
    --------------------------------------------------------------------------

Rotated components

    ----------------------------------------------------------
        Variable |    Comp1     Comp2     Comp3 | Unexplained
    -------------+------------------------------+-------------
          idade4 |   0.5468   -0.0806    0.0478 |       .4134
            sexo |  -0.0008    0.0010    0.9982 |     .002084
        igg_denv |   0.5583    0.0901   -0.0102 |        .417
       marital_2 |  -0.6161    0.0017    0.0291 |       .2878
        educacao |   0.0761   -0.6688   -0.0153 |       .4788
    income_che~2 |   0.0624    0.7336   -0.0089 |       .4039
    ----------------------------------------------------------

Component rotation matrix

    --------------------------------------------
                 |    Comp1     Comp2     Comp3
    -------------+------------------------------
           Comp1 |   0.9856   -0.1413    0.0925
           Comp2 |   0.1165    0.9654    0.2335
           Comp3 |  -0.1223   -0.2194    0.9679
    --------------------------------------------
```

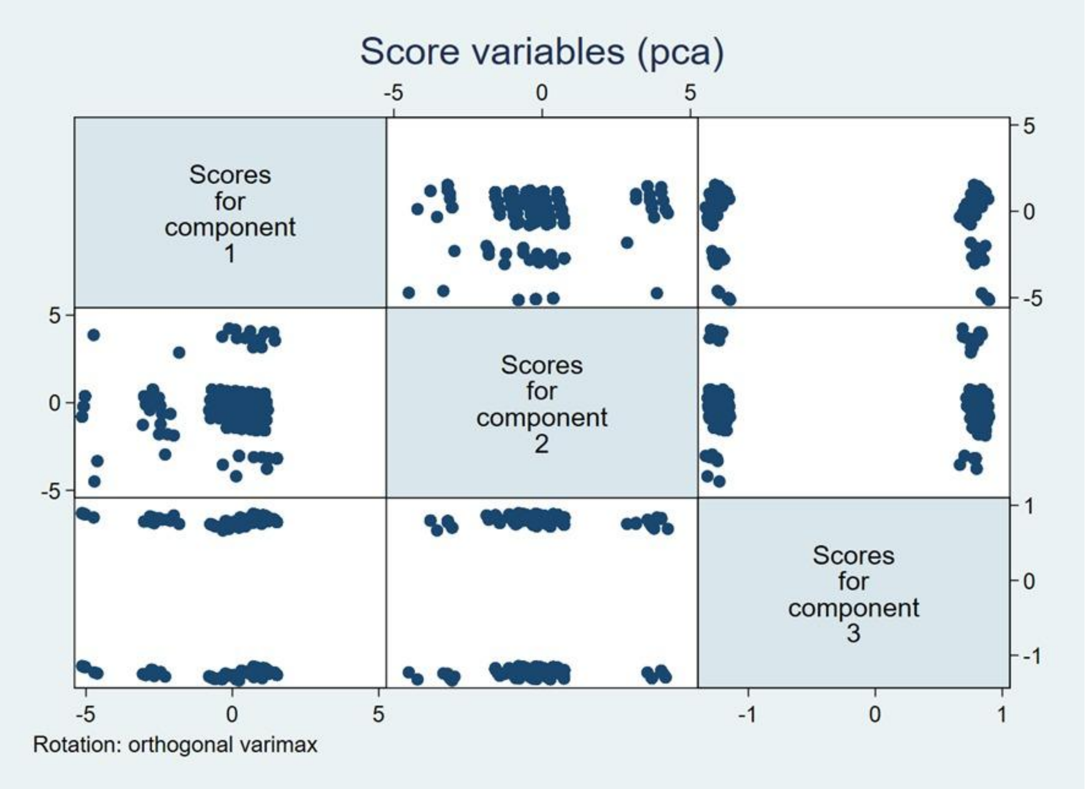

S1 Fig 1 - Individual Factor Scores from Principal Component Analysis (PCA) of Symptomatic Chikungunya Risk Factors

```
estat loading

Principal component loadings (unrotated)
    component normalization: sum of squares(column) = 1

    --------------------------------------------
                 |    Comp1     Comp2     Comp3
    -------------+------------------------------
          idade4 |    .5548  -.002979   -.00288
            sexo |   .09137      .234     .9661
        igg_denv |    .5366     .1496   -.09791
       marital_2 |   -.6048   -.06331     .1031
        educacao |    .1681    -.6403     .1226
    income_che~2 | -.04298     .7133    -.1772
    --------------------------------------------
```

Graph after rotation - loading components

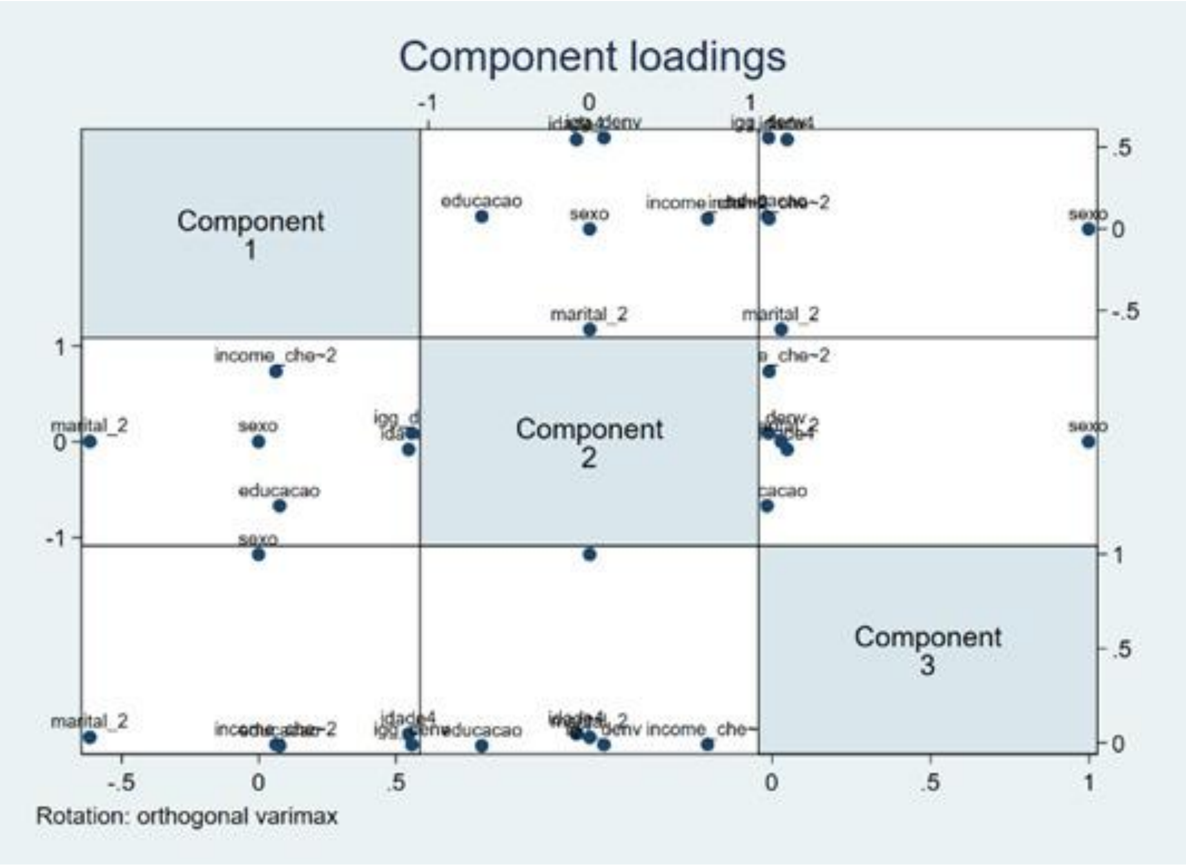

S1 Fig 2 - Varimax-Rotated PCA Loadings for Three Extracted Components.

```
predict fac1 fac2 fac3, score

Scoring coefficients for orthogonal varimax rotation
    sum of squares(column-loading) = 1

    --------------------------------------------
        Variable |    Comp1     Comp2     Comp3
    -------------+------------------------------
          idade4 |   0.5468   -0.0806    0.0478
            sexo |  -0.0008    0.0010    0.9982
        igg_denv |   0.5583    0.0901   -0.0102
       marital_2 |  -0.6161    0.0017    0.0291
        educacao |   0.0761   -0.6688   -0.0153
    income_che~2 |   0.0624    0.7336   -0.0089
    --------------------------------------------

summarize fac1 fac2 fac3

    Variable |        Obs        Mean    Std. Dev.       Min        Max
-------------+---------------------------------------------------------
        fac1 |        760   -1.37e-10    1.371639  -5.135303    1.52254
        fac2 |        760    6.76e-10    1.055533  -4.496763   4.235144
        fac3 |        760    9.41e-10     1.00079  -1.335995   .8951411
```

Bartlett's test of sphericity

```
estat smc

Squared multiple correlations of variables with all other variables

    -----------------------
        Variable |     smc
    -------------+---------
          idade4 |  0.2595
            sexo |  0.0055
        igg_denv |  0.2416
      marital_2 |  0.3509
        educacao |  0.0273
    income_che~2 |  0.0135
    -----------------------
```

```
alpha idade4 sexo igg_denv marital_2 educacao income_chef_
Test scale = mean(unstandardized items)
Reversed items:  marital_2 income_chef_2
Average interitem covariance:     .1540263
Number of items in the scale:            6
Scale reliability coefficient:      0.3872
```

Correlation between components

```
correlate fac1 fac2 fac3 (obs=760)

             |     fac1     fac2     fac3
-------------+---------------------------
        fac1 |   1.0000
        fac2 |  -0.0793   1.0000
        fac3 |   0.0633   0.0135   1.0000
```

Logistic regression of the association between the three components with the outcome (symptomatic chikungunya)

```
logistic chisint fac1 fac2 fac3

Logistic regression                             Number of obs     =        760
                                                LR chi2(3)        =      40.43
                                                Prob > chi2       =     0.0000
Log likelihood = -444.04091                     Pseudo R2         =     0.0435

------------------------------------------------------------------------------
     chisint | Odds Ratio   Std. Err.      z    P>|z|     [95% Conf. Interval]
-------------+----------------------------------------------------------------
        fac1 |   1.305948   .0742542     4.69   0.000     1.168229    1.459903
        fac2 |   1.161128   .0927951     1.87   0.062     .9927818    1.358021
        fac3 |   1.342429    .107474     3.68   0.000     1.147479      1.5705
       _cons |   2.428767   .1997903    10.79   0.000     2.067122    2.853683
------------------------------------------------------------------------------
Note: _cons estimates baseline odds.
```

```
logit chisint fac1 fac2 fac3, or level(95)

Iteration 0:   log likelihood = -464.25687
Iteration 1:   log likelihood = -444.21541
Iteration 2:   log likelihood = -444.04093
Iteration 3:   log likelihood = -444.04091
```

```
Logistic regression                               Number of obs     =        760
                                                  LR chi2(3)        =      40.43
                                                  Prob > chi2       =     0.0000
Log likelihood = -444.04091                       Pseudo R2         =     0.0435

------------------------------------------------------------------------------
     chisint | Odds Ratio   Std. Err.      z    P>|z|     [95% Conf. Interval]
-------------+----------------------------------------------------------------
        fac1 |   1.305948   .0742542     4.69   0.000     1.168229    1.459903
        fac2 |   1.161128   .0927951     1.87   0.062     .9927818    1.358021
        fac3 |   1.342429    .107474     3.68   0.000     1.147479      1.5705
       _cons |   2.428767   .1997903    10.79   0.000     2.067122    2.853683
------------------------------------------------------------------------------
Note: _cons estimates baseline odds.
```

<u>Model fit statistics</u>

```
estat ic

Akaike's information criterion and Bayesian information criterion

-----------------------------------------------------------------------------
       Model |        Obs  ll(null)  ll(model)      df         AIC        BIC
-------------+---------------------------------------------------------------
           . |        760 -464.2569  -444.0409       4    896.0818   914.6151
-----------------------------------------------------------------------------
               Note: N=Obs used in calculating BIC; see [R] BIC note.
```

```
Hosmer-Lemeshow test for goodness of fit.

estat gof, group(10)

Logistic model for chisint, goodness-of-fit test

  (Table collapsed on quantiles of estimated probabilities)

       number of observations =       760
             number of groups =        10
      Hosmer-Lemeshow chi2(8) =         8.11
                  Prob > chi2 =         0.4231
estat class

Logistic model for chisint

              -------- True --------
Classified |          D            ~D  |      Total
-----------+--------------------------+-----------
     +     |        513           198  |        711
     -     |         19            30  |         49
-----------+--------------------------+-----------
   Total   |        532           228  |        760
```

```
Classified + if predicted Pr(D) >= .5
True D defined as chisint != 0
--------------------------------------------------
Sensitivity                     Pr( +| D)   96.43%
Specificity                     Pr( -|~D)   13.16%
Positive predictive value       Pr( D| +)   72.15%
Negative predictive value       Pr(~D| -)   61.22%
--------------------------------------------------
False + rate for true ~D        Pr( +|~D)   86.84%
False - rate for true D         Pr( -| D)    3.57%
False + rate for classified +   Pr(~D| +)   27.85%
False - rate for classified -   Pr( D| -)   38.78%
--------------------------------------------------
Correctly classified                        71.45%
--------------------------------------------------
```

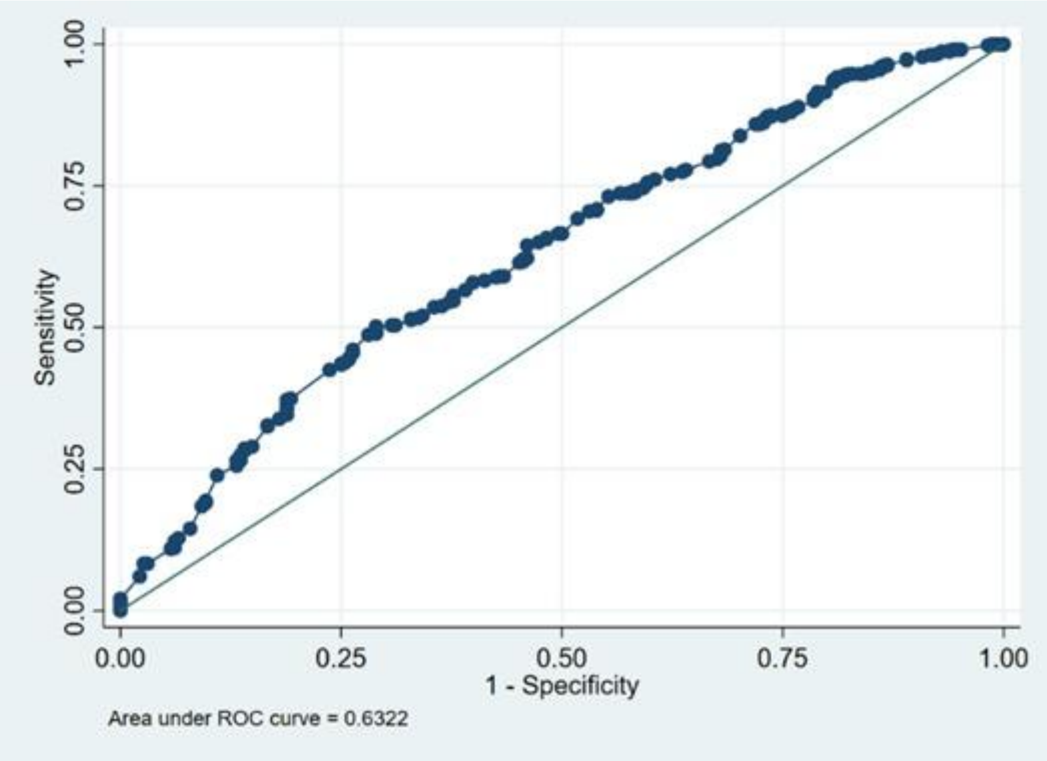

S1 Fig 3 - Receiver Operating Characteristic (ROC) Curve for the Logistic Regression Model Predicting Symptomatic Chikungunya

PCA – Chronic arthralgia

```
describe idade4 ieva income_chef_2 sexo marital_2 comorbidade

storage   display    value
variable name   type    format     label      variable label
------------------------------------------------------------------------------
idade4          byte    %9.0g                 RECODE of age (age)
ieva            float   %9.0g                 RECODE of eva
income_chef_2   float   %9.0g
sexo            byte    %10.0g                sexo
marital_2       float   %9.0g
comorbidade     float   %9.0g
```

```
          summarize idade4 ieva income_chef_2 sexo marital_2 comorbidade

    Variable |        Obs        Mean    Std. Dev.       Min        Max
-------------+---------------------------------------------------------
      idade4 |        499    2.793587    1.031375          1          4
        ieva |        499    .7234469    .4477424          0          1
income_che~2 |        486    1.654321    .6794873          0          8
        sexo |        499    .6613226    .4737349          0          1
   marital_2 |        499    .8056112    1.689885          0          8
-------------+---------------------------------------------------------
 comorbidade |        499    .3386774    .4737349          0          1
```

Sampling Adequacy Test

KMO and Bartlett tests

```
. factor idade4 ieva income_chef_2 sexo marital_2 comorbidade
(obs=486)

Factor analysis/correlation                      Number of obs    =    486
Method: principal factors                    Retained factors =          4
Rotation: (unrotated)                        Number of params =         15

--------------------------------------------------------------------------
Factor  |   Eigenvalue   Difference        Proportion   Cumulative
-------------+------------------------------------------------------------
Factor1  |      0.94430      0.61107            1.0634       1.0634
Factor2  |      0.33323      0.22299            0.3753       1.4387
Factor3  |      0.11024      0.10589            0.1241       1.5628
Factor4  |      0.00435      0.20775            0.0049       1.5677
Factor5  |     -0.20339      0.09733           -0.2290       1.3387
Factor6  |     -0.30073            .           -0.3387       1.0000
--------------------------------------------------------------------------
LR test: independent vs. saturated:  chi2(15) =  243.24 Prob>chi2 = 0.0000

Factor loadings (pattern matrix) and unique variances

    ----------------------------------------------------------------
        Variable |  Factor1   Factor2   Factor3   Factor4 |   Uniqueness
    -------------+----------------------------------------+--------------
          idade4 |   0.6453   -0.1366    0.0614    0.0128 |      0.5610
            ieva |   0.2690    0.2274   -0.1829   -0.0192 |      0.8421
    income_che~2 |  -0.1465    0.0426   -0.0731    0.0609 |      0.9677
            sexo |   0.1646    0.3826   -0.0764    0.0079 |      0.8206
       marital_2 |  -0.4662    0.2630    0.1562   -0.0034 |      0.6891
     comorbidade |   0.4355    0.2132    0.1935    0.0067 |      0.7274
    ----------------------------------------------------------------

. estat kmo

Kaiser-Meyer-Olkin measure of sampling adequacy

-----------------------
Variable |     kmo
-------------+---------
idade4 |  0.5041
ieva |  0.5929
income_che~2 |  0.6682
sexo |  0.5181
marital_2 |  0.4823
comorbidade |  0.4948
-------------+---------
Overall |  0.5094
-----------------------

. pca idade4 ieva income_chef_2 sexo marital_2 comorbidade
```

```
Principal components/correlation                 Number of obs    =        486
Number of comp.  =          6
Trace            =          6
Rotation: (unrotated = principal)           Rho              =     1.0000

--------------------------------------------------------------------------
Component |   Eigenvalue   Difference         Proportion   Cumulative
-------------+------------------------------------------------------------
Comp1 |      1.72172      .519075            0.2870       0.2870
Comp2 |      1.20264      .217881            0.2004       0.4874
Comp3 |      .984763     .0449872            0.1641       0.6515
Comp4 |      .939776      .238452            0.1566       0.8082
Comp5 |      .701324      .251552            0.1169       0.9250
Comp6 |      .449772            .            0.0750       1.0000
--------------------------------------------------------------------------

Principal components (eigenvectors)

         ------------------------------------------------------------------------------
     Variable |    Comp1     Comp2     Comp3     Comp4     Comp5     Comp6 | Unexplained
          -------------+------------------------------------------------------------+--
       idade4 |   0.6023   -0.2783    0.0883    0.2316   -0.0215    0.7057 |           0
         ieva |   0.3422    0.4172    0.1527   -0.6024    0.5639    0.0682 |           0
  income_che~2 |  -0.1881    0.1936    0.8419    0.4241    0.1963   -0.0016 |           0
         sexo |   0.2239    0.7031    0.0430    0.0037   -0.6709    0.0592 |           0
    marital_2 |  -0.4630    0.4171   -0.4049    0.2689    0.3050    0.5314 |           0
   comorbidade |   0.4693    0.2066   -0.3071    0.5756    0.3161   -0.4599 |           0
 --------------------------------------------------------------------------------------

rotate, varimax

Principal components/correlation                 Number of obs    =      486
Number of comp.  =          6
Trace            =          6
Rotation: orthogonal varimax (Kaiser off)    Rho              =     1.0000

--------------------------------------------------------------------------
Component |     Variance   Difference         Proportion   Cumulative
-------------+------------------------------------------------------------
Comp1 |           1  1.17244e-06            0.1667       0.1667
Comp2 |           1  7.69945e-07            0.1667       0.3333
Comp3 |           1  1.97404e-07            0.1667       0.5000
Comp4 |           1  1.52103e-07            0.1667       0.6667
Comp5 |           1  1.77356e-06            0.1667       0.8333
Comp6 |      .999998            .            0.1667       1.0000
--------------------------------------------------------------------------

Rotated components

  ------------------------------------------------------------------------------- Variable
              |    Comp1     Comp2     Comp3     Comp4     Comp5     Comp6 | Unexplained
         -------------+-------------------------------------------------------------+---
       idade4 |   1.0000    0.0000   -0.0000   -0.0000    0.0000    0.0000 |           0
         ieva |  -0.0000    1.0000    0.0000    0.0000   -0.0000    0.0000 |           0
  income_che~2 |   0.0000   -0.0000    1.0000   -0.0000   -0.0000   -0.0000 |           0
         sexo |  -0.0000    0.0000    0.0000   -0.0000    1.0000    0.0000 |           0
    marital_2 |  -0.0000   -0.0000    0.0000   -0.0000   -0.0000    1.0000 |           0
   comorbidade |   0.0000   -0.0000    0.0000    1.0000    0.0000    0.0000 |           0
         ------------------------------------------------------------------------------
Component rotation matrix

--------------------------------------------------------------------------
|    Comp1      Comp2      Comp3      Comp4      Comp5      Comp6
-------------+------------------------------------------------------------
Comp1 |   0.6023     0.3422    -0.1881     0.4693     0.2239    -0.4630
Comp2 |  -0.2783     0.4172     0.1936     0.2066     0.7031     0.4171
Comp3 |   0.0883     0.1527     0.8419    -0.3071     0.0430    -0.4049
```

```
Comp4 |   0.2316   -0.6024    0.4241    0.5756    0.0037    0.2689
Comp5 |  -0.0215    0.5639    0.1963    0.3161   -0.6709    0.3050
Comp6 |   0.7057    0.0682   -0.0016   -0.4599    0.0592    0.5314
--------------------------------------------------------------------------
```

estat loading

Principal component loadings component normalization: sum of squares(column)= 1

	Comp1	Comp2	Comp3	Comp4	Comp5	Comp6
idade4	.6023	-.2783	.08829	.2316	-.02145	.7057
ieva	.3422	.4172	.1527	-.6024	.5639	.06822
income_che~2	-.1881	.1936	.8419	.4241	.1963	-.001627
sexo	.2239	.7031	.04302	.003705	-.6709	.05919
marital_2	-.463	.4171	-.4049	.2689	.305	.5314
comorbidade	.4693	.2066	-.3071	.5756	.3161	-.4599

predict fac1 fac2 fac3, score (3 components skipped)

Scoring coefficients for orthogonal varimax rotation
sum of squares(column-loading) = 1

Variable	Comp1	Comp2	Comp3	Comp4	Comp5	Comp6
idade4	1.0000	0.0000	-0.0000	-0.0000	0.0000	0.0000
ieva	-0.0000	1.0000	0.0000	0.0000	-0.0000	0.0000
income_che~2	0.0000	-0.0000	1.0000	-0.0000	-0.0000	-0.0000
sexo	-0.0000	0.0000	0.0000	-0.0000	1.0000	0.0000
marital_2	-0.0000	-0.0000	0.0000	-0.0000	-0.0000	1.0000
comorbidade	0.0000	-0.0000	0.0000	1.0000	0.0000	0.0000

summarize fac1 fac2 fac3

Variable	Obs	Mean	Std. Dev.	Min	Max
fac1	486	-2.51e-09	1.000001	-1.743484	1.16831
fac2	486	7.36e-10	1	-1.619098	.6163611
fac3	486	4.91e-10	1	-2.434661	9.338923

estat smc

Squared multiple correlations of variables with all other variables

Variable	smc
idade4	0.3093
ieva	0.0795
income_che~2	0.0174
sexo	0.0840
marital_2	0.2145
comorbidade	0.1833

logistic chikcronica fac1 fac2 fac3

```
Logistic regression                          Number of obs     =        486
                                             LR chi2(3)        =      94.24
                                             Prob > chi2       =     0.0000
Log likelihood = -272.14709                  Pseudo R2         =     0.1476
```

```
------------------------------------------------------------------------------
 chikcronica | Odds Ratio   Std. Err.      z    P>|z|     [95% Conf. Interval]
-------------+----------------------------------------------------------------
        fac1 |    1.96187   .2246512     5.89   0.000     1.567473    2.455501
        fac2 |   2.127722   .2734857     5.87   0.000     1.653888    2.737308
        fac3 |   .7803637   .0867602    -2.23   0.026     .6275685    .9703602
       _cons |   .4828738   .0541238    -6.49   0.000     .3876368    .6015091
------------------------------------------------------------------------------
Note: _cons estimates baseline odds.
```

```
logit chikcronica fac1 fac2 fac3, or level(95)

Iteration 0:   log likelihood = -319.26919
Iteration 1:   log likelihood = -273.77191
Iteration 2:   log likelihood = -272.15014
Iteration 3:   log likelihood = -272.14709
Iteration 4:   log likelihood = -272.14709

Logistic regression                             Number of obs     =        486
LR chi2(3)        =      94.24
Prob > chi2       =     0.0000
Log likelihood = -272.14709                     Pseudo R2         =     0.1476

------------------------------------------------------------------------------
chikcronica | Odds Ratio   Std. Err.      z    P>|z|     [95% Conf. Interval]
-------------+----------------------------------------------------------------
fac1 |    1.96187   .2246512     5.89   0.000     1.567473    2.455501
fac2 |   2.127722   .2734857     5.87   0.000     1.653888    2.737308
fac3 |   .7803637   .0867602    -2.23   0.026     .6275685    .9703602
_cons |   .4828738   .0541238    -6.49   0.000     .3876368    .6015091
------------------------------------------------------------------------------
Note: _cons estimates baseline odds.

estat ic

Akaike's information criterion and Bayesian information criterion

-----------------------------------------------------------------------------
Model |        Obs  ll(null)  ll(model)      df         AIC        BIC
-------------+---------------------------------------------------------------
. |        486 -319.2692  -272.1471       4    552.2942    569.039
-----------------------------------------------------------------------------
Note: N=Obs used in calculating BIC; see [R] BIC note.

estat gof, group(10)

Logistic model for chikcronica, goodness-of-fit test

(Table collapsed on quantiles of estimated probabilities)

number of observations =       486
number of groups =        10
Hosmer-Lemeshow chi2(8) = 13.45
Prob > chi2 =         0.0973

estat class

Logistic model for chikcronica

-------- True --------
Classified |         D            ~D  |      Total
-----------+--------------------------+-----------
+     |       87           59  |       146
-     |       91          249  |       340
-----------+--------------------------+-----------
Total   |       178          308  |       486

Classified + if predicted Pr(D) >= .5
True D defined as chikcronica != 0
--------------------------------------------------
Sensitivity                     Pr( +| D)   48.88%
Specificity                     Pr( -|~D)   80.84%
Positive predictive value       Pr( D| +)   59.59%
Negative predictive value       Pr(~D| -)   73.24%
--------------------------------------------------
```

```
False + rate for true ~D        Pr( +|~D)   19.16%
False - rate for true D         Pr( -| D)   51.12%
False + rate for classified +   Pr(~D| +)   40.41%
False - rate for classified -   Pr( D| -)   26.76%
--------------------------------------------------
Correctly classified                        69.14%
--------------------------------------------------
```

```
poisson chikcronica i.idade4 ieva i.income_chef_2 sexo, irr vce(robust)

Iteration 0:   log pseudolikelihood = -330.60385
Iteration 1:   log pseudolikelihood = -330.50504
Iteration 2:   log pseudolikelihood = -330.50475
Iteration 3:   log pseudolikelihood = -330.50475

Poisson regression                              Number of obs     =        499
                                                Wald chi2(8)      =      77.90
                                                Prob > chi2       =     0.0000
Log pseudolikelihood = -330.50475               Pseudo R2         =     0.0959

------------------------------------------------------------------------------
             |               Robust
 chikcronica |        IRR   Std. Err.      z    P>|z|     [95% Conf. Interval]
-------------+----------------------------------------------------------------
      idade4 |
          2  |    2.38696   .9943512     2.09   0.037     1.055005     5.40052
          3  |   4.098831   1.638744     3.53   0.000     1.872161    8.973811
          4  |   4.590005   1.831472     3.82   0.000     2.099775    10.03353
             |
        ieva |   2.911848   .6632066     4.69   0.000     1.863365    4.550296
             |
income_chef_2 |
          1  |   .8287348   .1226013    -1.27   0.204     .6201412    1.107492
          2  |   .6886125   .0891593    -2.88   0.004     .5342741    .8875353
          8  |   .6573483    .262903    -1.05   0.294     .3001663    1.439558
             |
        sexo |   1.397455   .1926017     2.43   0.015     1.066651    1.830851
       _cons |   .0465737   .0217639    -6.56   0.000     .0186369    .1163879
------------------------------------------------------------------------------
Note: _cons estimates baseline incidence rate.

mdesc idade4 ieva income_chef_2 sexo chikcronica

    Variable    |     Missing          Total     Percent Missing
----------------+-----------------------------------------------
         idade4 |           0            499           0.00
           ieva |           0            499           0.00
   income_che~2 |           0            499           0.00
           sexo |           0            499           0.00
    chikcronica |           0            499           0.00
----------------+-----------------------------------------------

estat gof

         Deviance goodness-of-fit =  297.0095
         Prob > chi2(490)         =    1.0000

         Pearson goodness-of-fit  =    328.33
         Prob > chi2(490)         =    1.0000
```

```
poisson chikcronica i.idade4 ieva i.income_chef_2 sexo, irr vce(robust)

Iteration 0:   log pseudolikelihood = -322.55177
Iteration 1:   log pseudolikelihood = -322.46645
```

```
Iteration 2:   log pseudolikelihood = -322.46617
Iteration 3:   log pseudolikelihood = -322.46617

Poisson regression                              Number of obs     =        485
                                                Wald chi2(7)      =      78.10
                                                Prob > chi2       =     0.0000
Log pseudolikelihood = -322.46617               Pseudo R2         =     0.0953

------------------------------------------------------------------------------
             |               Robust
 chikcronica |        IRR   Std. Err.      z    P>|z|     [95% Conf. Interval]
-------------+----------------------------------------------------------------
      idade4 |
          2  |   2.250229   .9409221     1.94   0.052     .9915189    5.106842
          3  |   4.044009   1.615754     3.50   0.000      1.84808    8.849187
          4  |   4.483465   1.787552     3.76   0.000     2.052299    9.794601
             |
        ieva |   2.762166   .6252216     4.49   0.000     1.772469    4.304482
             |
income_chef_2 |
          1  |   .8274172   .1229577    -1.27   0.202     .6183471    1.107176
          2  |   .6876143   .0895726    -2.88   0.004     .5326753    .8876204
             |
        sexo |   1.426827   .1981761     2.56   0.010      1.08679    1.873256
       _cons |   .0493438   .0229389    -6.47   0.000     .0198394    .1227263
------------------------------------------------------------------------------
Note: _cons estimates baseline incidence rate.


.  estat gof

         Deviance goodness-of-fit =  297.0095
         Prob > chi2(490)         =    1.0000

         Pearson goodness-of-fit  =    328.33
         Prob > chi2(490)         =    1.0000
```

Conclusion: The full model showed a slightly lower (more negative) log pseudolikelihood value, indicating a marginally better fit to the data. The difference in Pseudo R^2 is minimal (0.0959 vs. 0.0953), suggesting that the exclusion of missing cases does not significantly impact the overall explanatory power of the model.

Checking missing values before imputation

```
 mdesc idade4 ieva income_cat sexo chikcronica

    Variable    |     Missing          Total     Percent Missing
----------------+-----------------------------------------------
         idade4 |           0            499           0.00
           ieva |           0            499           0.00
     income_cat |          14            499           2.81
           sexo |           0            499           0.00
    chikcronica |           0            499           0.00
----------------+-----------------------------------------------


mi register regular idade4 ieva sexo chikcronica
mi register imputed income_chef_2
(14 m=0 obs. now marked as incomplete)

mi impute chained (ologit) income_chef_2 = idade4 ieva sexo chikcronica, add(5)
note: missing-value pattern is monotone; no iteration performed

Conditional models (monotone):
```

```
        income_cat: ologit income_cat idade4 ieva sexo chikcronica

Performing chained iterations ...

Multivariate imputation                    Imputations =        5
Chained equations                                added =        5
Imputed: m=1 through m=5                       updated =        0

Initialization: monotone                    Iterations =        0
                                               burn-in =        0
        income_cat: ordered logistic regression

------------------------------------------------------------------
                   |               Observations per m
                   |----------------------------------------------
          Variable |   Complete   Incomplete   Imputed |     Total
-------------------+-----------------------------------+----------
        income_cat |        485           14        14 |       499
------------------------------------------------------------------
(complete + incomplete = total; imputed is the minimum across m
 of the number of filled-in observations.)
```

```
mi estimate: poisson chikcronica i.idade4 ieva i.income_cat sexo, irr
vce(robust)

Multiple-imputation estimates                   Imputations       =          5
Poisson regression                              Number of obs     =        499
                                                Average RVI       =     0.0139
                                                Largest FMI       =     0.0618
DF adjustment:   Large sample                   DF:     min       =   1,106.23
                                                        avg       =   2.81e+08
                                                        max       =   7.87e+08
Model F test:       Equal FMI                   F(   7,96612.0)   =      10.92
Within VCE type:       Robust                   Prob > F          =     0.0000

------------------------------------------------------------------------------
 chikcronica |      Coef.   Std. Err.      t    P>|t|     [95% Conf. Interval]
-------------+----------------------------------------------------------------
      idade4 |
          2  |   .8698679   .4169394     2.09   0.037     .0526818    1.687054
          3  |   1.411923   .4000766     3.53   0.000      .627787    2.196058
          4  |   1.525718   .3991266     3.82   0.000     .7434442    2.307992
             |
        ieva |   1.070535   .2266381     4.72   0.000     .6263328    1.514738
             |
  income_cat |
          1  |  -.1898225   .1524914    -1.24   0.213    -.4889907    .1093457
          2  |  -.3684566   .1340287    -2.75   0.006    -.6314359   -.1054774
             |
        sexo |   .3329586   .1372593     2.43   0.015     .0639353    .6019819
       _cons |  -3.073924   .4685762    -6.56   0.000    -3.992323   -2.155525
------------------------------------------------------------------------------
```
